# Explainable machine learning for breast mass characterization and malignancy risk stratification: multimodal integration of AI-derived structured digital breast tomosynthesis features and peripheral blood immune-inflammatory biomarkers

**DOI:** 10.3389/fcell.2026.1917987

**Published:** 2026-09-03

**Authors:** Lu Nie, Yun Wei, Xinyu Feng, Yizi Lu

**Affiliations:** Department of Radiology, Changzhou Maternal and Child Health Care Hospital, Changzhou Medical Center, Nanjing Medical University, Changzhou, China

**Keywords:** artificial intelligence, breast mass, digital breast tomosynthesis, machine learning, peripheral blood inflammatory indices, risk stratification, shap

## Abstract

**Background:**

Accurate discrimination between benign and malignant breast masses is essential for biopsy decisions and individualized management. This study aimed to develop an interpretable machine-learning model integrating artificial intelligence (AI)-structured digital breast tomosynthesis (DBT) mass features with peripheral blood immune-inflammatory indices.

**Methods:**

This retrospective study included 382 patients with 401 pathologically confirmed breast mass lesions (299 benign and 102 malignant) who underwent DBT before pathological examination. AI-structured DBT descriptors, clinical variables, and hematologic inflammatory indices were collected. Lesions were divided into training and testing cohorts using stratified random sampling at a 7:3 ratio. Least absolute shrinkage and selection operator (LASSO) logistic regression was used for feature selection, and five machine-learning models were trained. Model performance was evaluated using the area under the receiver operating characteristic curve (AUROC), calibration, decision curve analysis, Brier score, and confusion matrix. SHapley Additive exPlanations (SHAP) analysis was used to interpret the final model, and predicted probabilities were used for descriptive risk stratification.

**Results:**

Logistic LASSO regression selected nine predictors: suspicious calcifications, irregular mass shape, age, long-axis diameter, neutrophil-to-lymphocyte ratio (NLR), short-axis diameter, oval mass shape, absence of calcifications, and obscured margin. In the testing cohort, the logistic regression model achieved the highest AUROC (0.884, 95% CI: 0.807–0.950), with an accuracy of 0.860, sensitivity of 0.806, specificity of 0.878, and negative predictive value of 0.929. SHAP analysis results indicate that suspicious calcifications, advanced age, irregular morphology, elevated NLR, and larger lesion size may be the primary factors contributing to the prediction of malignancy. The malignancy rate observed in this study ranged from 2.5% in the low-risk group to 67.6% in the high-risk group.

**Conclusion:**

The logistic regression (LR) model integrating AI-derived structured DBT features, clinical variables, and peripheral blood immune-inflammatory biomarkers demonstrated favorable performance for breast mass classification and malignancy risk stratification, with a test-set AUROC of 0.884. Prospective multicentre external validation is required.

## 1 Introduction

Breast cancer is one of the most common malignant tumors among women, accounting for approximately one-quarter of all female cancer cases. Therefore, early detection and accurate diagnosis are crucial for improving patient prognosis (Bray et al., 2024). In particular, breast masses are among the most common abnormalities observed in breast imaging. Consequently, distinguishing between benign and malignant masses directly influences subsequent treatment plans, which may include biopsy, short-term imaging follow-up, and surgical treatment (Sanmugasiva et al., 2020).

Digital Breast Tomosynthesis (DBT) is a technique that involves multi-angle image acquisition and tomographic reconstruction to generate cross-sectional images from reconstructed breast volume data. Compared to Full-Field Digital Mammography (FFDM), where tissue overlap can cause issues, DBT improves lesion clarity and diagnostic accuracy. As DBT has gradually been adopted in clinical practice, it has been demonstrated to reduce recall rates and improve breast cancer detection rates, while also significantly enhancing the visualization of lesion margins, morphology, density, and relationships with adjacent structures (Friedewald et al., 2014; Morgan et al., 2005; Partyka et al., 2014). However, digital breast tomosynthesis (DBT) generates a large volume of images, and the interpretation process is time-consuming and dependent on the reader’s experience. Therefore, improving the objectivity of DBT feature extraction and the consistency of diagnostic evaluation has become an important research direction in breast imaging.

Artificial intelligence (AI), including machine-learning methods, can extract latent diagnostic information from complex clinical, imaging, and biological data (Benzekry, 2020). AI has been widely investigated for tumor diagnosis, treatment-response prediction, and prognostic assessment, including breast cancer imaging, and has achieved performance comparable to expert interpretation in selected diagnostic tasks (Bejnordi et al., 2017; McKinney et al., 2020).

The development and progression of breast cancer are not limited to local morphologic changes but are also associated with the tumor microenvironment and systemic immune-inflammatory status (Fridman et al., 2017; Hegde and Chen, 2020; Xie et al., 2023). Peripheral blood inflammatory indices, including the neutrophil-to-lymphocyte ratio (NLR), platelet-to-lymphocyte ratio (PLR), lymphocyte-to-monocyte ratio (LMR), systemic immune-inflammation index (SII), systemic inflammation response index (SIRI), and pan-immune-inflammation value (PIV), can partly reflect host inflammatory and immune status. Because these indices are derived from routine blood tests, they are inexpensive, readily available, and clinically accessible (Chen et al., 2020; Lee et al., 2021).

Accordingly, integrating AI-structured DBT mass features with peripheral blood immune-inflammatory indices may improve the discrimination of benign and malignant breast masses by combining local imaging information with systemic host-response information. Machine-learning approaches are particularly suited for multivariable integration, nonlinear modeling, and individualized risk prediction (Silva et al., 2022). This study aimed to construct an interpretable machine-learning model based on AI-structured DBT mass features, clinical variables, and peripheral blood immune-inflammatory indices for breast mass classification and malignancy risk stratification.

## 2 Materials and methods

### 2.1 Study design and patients

In this retrospective study, consecutive patients who presented to Changzhou Maternal and Child Healthcare Hospital between September 2024 and September 2025 were screened. Patients were eligible for inclusion if they met the following criteria: (1) age >18 years; (2) completion of digital breast tomosynthesis before pathological examination; and (3) the presence of clearly identifiable breast mass lesions on digital breast tomosynthesis images. The exclusion criteria were as follows: (1) concurrent non-breast malignant tumors; (2) a history of breast surgery, radiotherapy, or chemotherapy; (3) pregnancy; (4) incomplete imaging data or motion artifacts affecting lesion characterization, such as poor edge sharpness (sharpness score <50%); and (5) acute infection, active inflammatory disease, hematologic disorders, or other conditions that may affect peripheral blood immune-inflammatory markers. A total of 382 patients were enrolled, comprising 401 lesions, of which 299 were benign and 102 were histopathologically confirmed as malignant. Histopathology served as the reference standard, and breast cancer was defined as a positive outcome. Because eligibility required a pathological diagnosis, this cohort represents a diagnostically enriched population and may not fully represent screening or follow-up populations. The study protocol was approved by the Ethics Committee of Changzhou Maternal and Child Healthcare Hospital, and informed consent was waived (approval no. 2026-K016-01).

### 2.2 DBT imaging and AI-structured reporting

DBT was performed using a GE Senographe Pristina digital breast tomosynthesis system. Craniocaudal (CC) and mediolateral oblique (MLO) views were acquired for all patients. A step-and-shoot acquisition mode was used, in which the X-ray tube remained stationary during each exposure and data acquisition. The scanning angle was 25° (±12.5°), nine exposures were obtained, and the mean scanning time was <10 s. Images were postprocessed using an adaptive statistical iterative reconstruction platform to generate thin-slice images, slab images, and two-dimensional synthesized view preview images.

**FIGURE 1:**
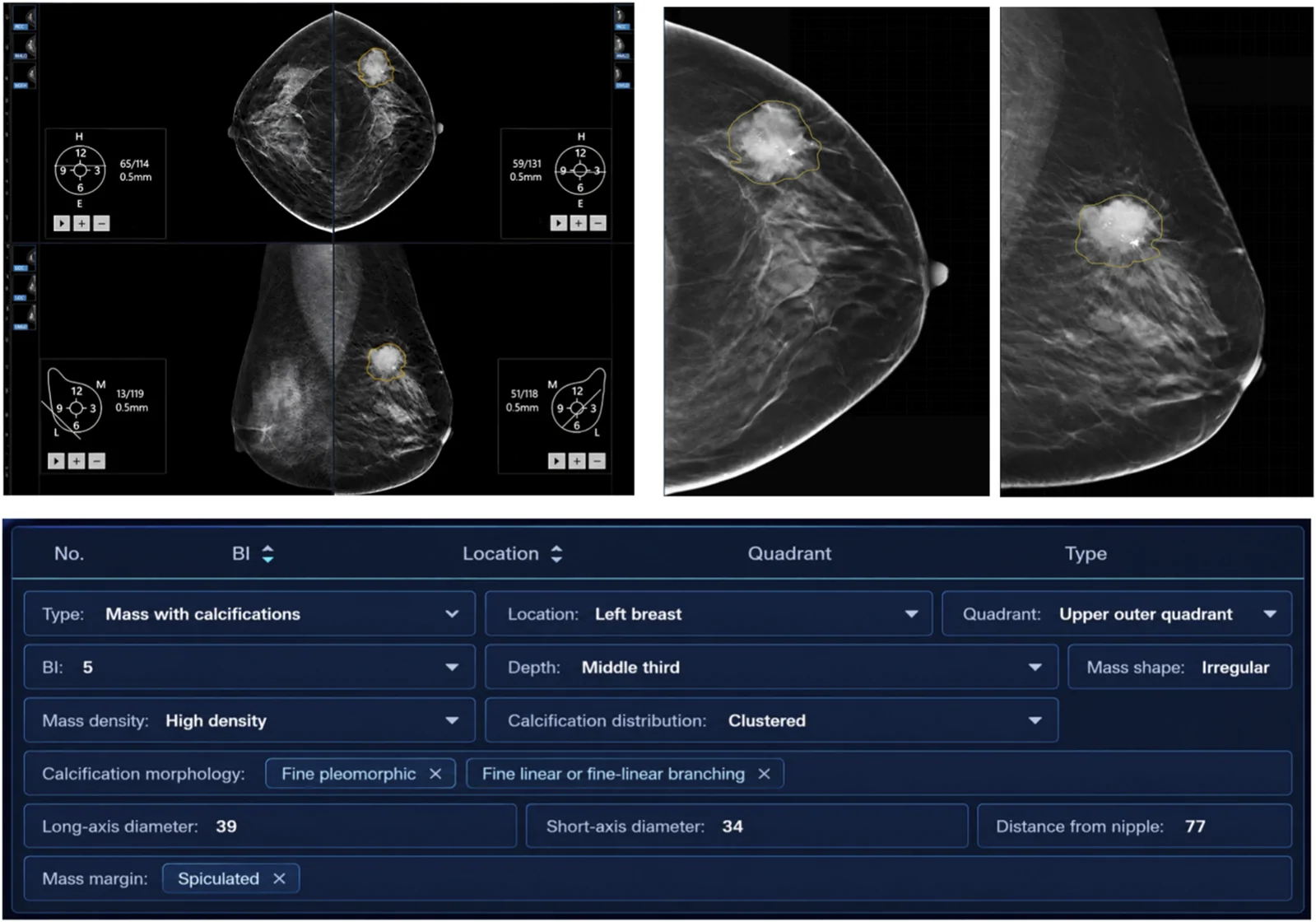
Representative breast cancer case analyzed using the AI-assisted diagnostic system. The upper panels show digital breast tomosynthesis images with automated lesion localization, and the lower panel shows the AI-structured graphic report summarizing BI-RADS-related descriptors, including lesion location, mass shape, density, margin, calcification features, and lesion size. The lesion was *pathologically* confirmed as breast cancer.

An intelligent breast AI-assisted diagnostic system developed by Beijing Deepwise and League of PHD Technology Co., Ltd. was used. The system had received Class III medical device registration approval from the National Medical Products Administration and was applicable to individuals aged 18 years or older. Based on the fifth edition of the American College of Radiology Breast Imaging Reporting and Data System (BI-RADS) lexicon (D’Orsi et al., 2013; Sedgwick et al., 2015), the system automatically analyzed lesion attributes, including mass margin, shape, density, calcification distribution, and calcification morphology. BI-RADS categories were then generated according to lesion morphology, and an AI-structured graphic report was produced. A representative breast cancer case with AI-assisted lesion analysis and the corresponding AI-structured graphic report is shown in Figure 1.

### 2.3 Peripheral blood immune-inflammatory indices

Complete blood count results obtained within 24 h after admission were extracted from the electronic medical record system. The extracted hematologic variables included neutrophil count, lymphocyte count, monocyte count, and platelet count. Peripheral blood immune-inflammatory indices were then calculated as follows: NLR = neutrophil count/lymphocyte count; PLR = platelet count/lymphocyte count; LMR = lymphocyte count/monocyte count; SII = platelet count x neutrophil count/lymphocyte count; SIRI = neutrophil count x monocyte count/lymphocyte count; and PIV = neutrophil count x platelet count x monocyte count/lymphocyte count.

### 2.4 Machine-learning model development and evaluation

Only continuous predictors, including age, lesion diameters, and peripheral blood immune-inflammatory indices, were standardized using Z-score transformation to reduce scale-related effects during model training. Categorical predictors were not standardized; they were converted into dummy variables, with the most common or clinically conventional category used as the reference. For example, mass shape and mass margin were represented by separate indicator variables for their respective categories. Lesions were used as the unit of analysis. All lesions were divided into training and testing cohorts using stratified random sampling at a 7:3 ratio to maintain similar proportions of benign and malignant lesions in both cohorts. The primary split was performed at the lesion level. Model training, feature selection, and hyperparameter optimization were performed only in the training cohort, whereas the testing cohort was reserved for independent performance evaluation. To assess the possible effect of multiple lesions contributed by the same patient, an additional patient-grouped sensitivity analysis was performed. Patient identity was used as the grouping variable, all lesions from the same patient were assigned to the same development or testing cohort, and patient-grouped five-fold cross-validation was used during model tuning.

The candidate features were derived from AI-structured DBT imaging descriptors, clinical variables, and peripheral blood immune-inflammatory markers. Least absolute shrinkage and selection operator (LASSO) logistic regression was first used for feature selection. Five-fold cross-validation was used to determine the optimal regularization parameter, and variables with nonzero coefficients were retained for subsequent model building. Based on the selected features, five machine-learning models were constructed: logistic regression (LR), random forest (RF), decision tree (DT), support vector machine (SVM), and XGBoost. Hyperparameters were optimized using five-fold grid search within the training cohort. The principal parameters included regularization strength for LR; tree number, depth, and minimum split or leaf size for RF and DT; C, kernel, and gamma for SVM; and n_estimators, max_depth, learning_rate, subsample, and colsample_bytree for XGBoost. The parameter combination yielding the highest mean cross-validation AUROC was retained for each model.

**FIGURE 2:**
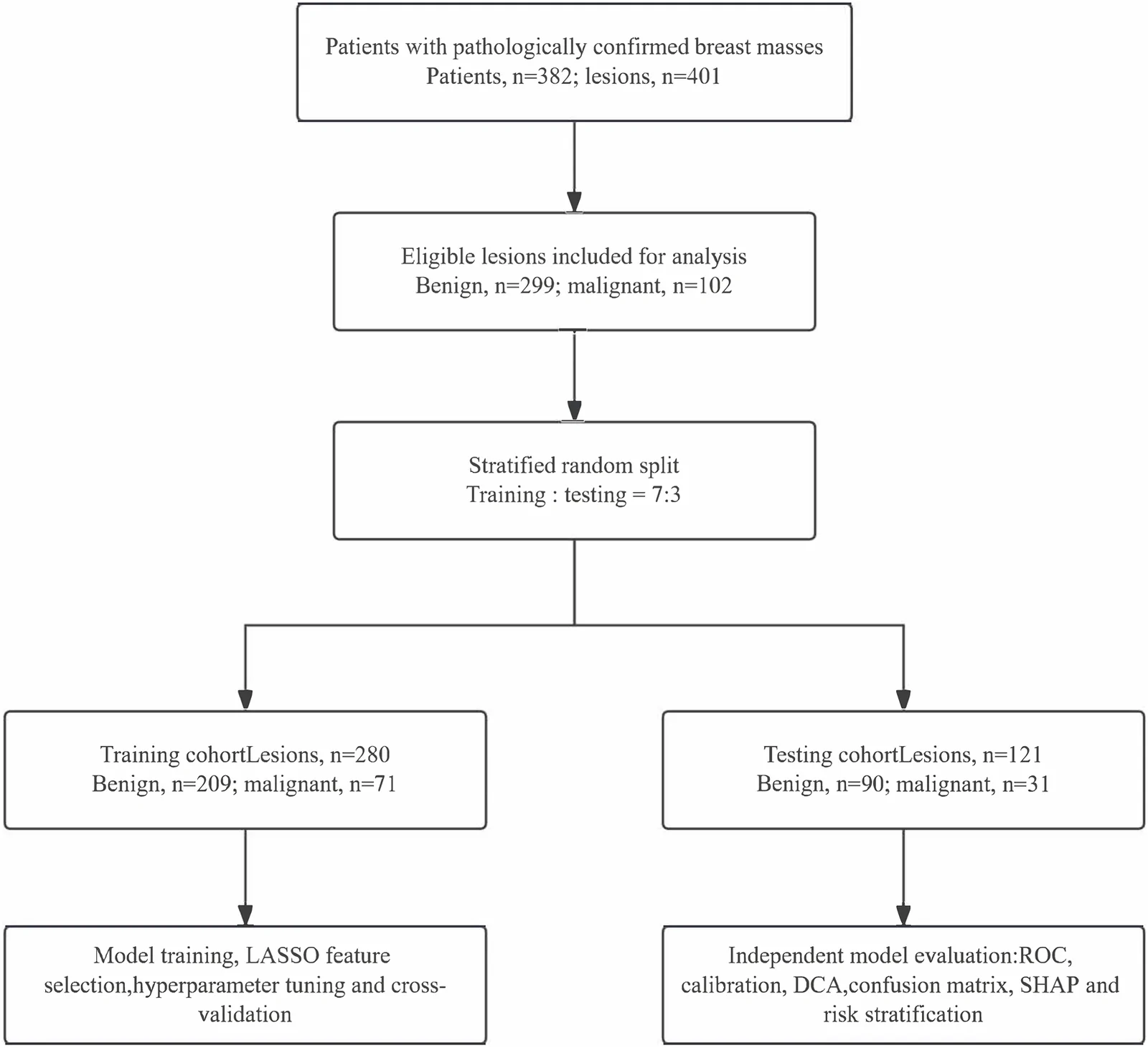
Workflow of multimodal integration and explainable machine-learning modeling for breast mass characterization and malignancy risk stratification. Multimodal features included artificial intelligence-derived structured digital breast tomosynthesis features, clinical information, and peripheral blood immune-inflammatory biomarkers. The training cohort was used for feature selection and model development, whereas the testing cohort was used for independent evaluation. The final model was further interpreted using SHAP analysis and applied for malignancy risk stratification.

The area under the receiver operating characteristic curve (AUROC) was used as the primary discrimination metric. Brier score, calibration curves, decision curve analysis (DCA), and confusion matrices were jointly used to evaluate probabilistic accuracy, calibration, clinical net benefit, and classification performance. The final model was selected by considering testing-cohort discrimination, Brier score and calibration, DCA, classification metrics, parsimony, clinical applicability, and interpretability rather than AUROC alone. SHAP analysis was conducted to quantify the contribution of each feature to malignancy prediction. A positive SHAP value indicates an increased probability of malignant prediction, whereas a negative SHAP value indicates a decreased probability. The analysis was prediction-focused; conventional post-selection Wald statistics and p-values were not used to rank predictors. For descriptive clinical interpretation, predicted probabilities from the testing cohort were divided into low-, intermediate-, and high-risk groups based on tertiles. These data-dependent groups were intended only to illustrate a risk gradient and were not prespecified clinical decision thresholds. The overall workflow is shown in Figure 2.

For the supplementary analyses, AUROC confidence intervals in the patient-grouped testing cohort were estimated using 3,000 patient-cluster bootstrap resamples. Pairwise DeLong tests were used to compare the LR AUROC with those of RF, XGBoost, SVM, and DT, and Holm adjustment was applied across the four LR-versus-comparator comparisons. Because conventional DeLong inference is lesion-level and not cluster-robust, these P values were considered supportive and interpreted cautiously. Spearman rank correlations among NLR, PLR, LMR, SII, PIV, and SIRI and the bootstrap LASSO stability analysis were restricted to the patient-grouped development cohort to avoid using the testing cohort for feature assessment. Feature-selection stability was evaluated in 1,000 patient-level bootstrap resamples; all lesions from a sampled patient were retained together, and the frequency with which each inflammatory index received a nonzero LASSO coefficient was recorded.

### 2.5 Statistical analysis

All statistical analyses were performed using Python 3.13.5 with NumPy 2.3.5, pandas 2.2.3, SciPy 1.17.0, scikit-learn 1.8.0, XGBoost 3.1.3, SHAP 0.50.0, statsmodels 0.14.6, and Matplotlib 3.10.8. Continuous variables were first assessed for normality. Normally distributed variables were expressed as mean ± standard deviation and compared using the independent-samples t-test. Non-normally distributed variables were expressed as median and interquartile range and compared using the Mann-Whitney U test. Categorical variables were expressed as counts and percentages and compared using the chi-square test or Fisher exact test, as appropriate. All tests were two-sided, and P < 0.05 was considered statistically significant.

## 3 Results

### 3.1 Patient characteristics and univariable analysis

A total of 382 patients with 401 breast mass lesions were included. Among these lesions, 299 were benign and 102 were malignant. Patients in the malignant group were older than those in the benign group [54 (46, 62) years vs. 41 (36, 48) years; P < 0.001]. Lesion side did not differ significantly between the two groups (P = 0.179). In contrast, BI-RADS category, lesion depth, mass shape, mass density, long-axis diameter, short-axis diameter, mass margin, and calcification status differed significantly between benign and malignant lesions.

Malignant lesions more frequently showed higher BI-RADS categories, irregular shape, high density, noncircumscribed margins, and suspicious calcifications. Compared with the reference categories, BI-RADS 5 (odds ratio [OR] = 119.7, 95% confidence interval [CI]: 25.910–553.1), irregular mass shape (OR = 19.572, 95% CI: 10.993–34.845), high density (OR = 17.165, 95% CI: 9.841–29.938), spiculated margin (OR = 40.000, 95% CI: 4.617–346.6), and suspicious calcifications (OR = 38.043, 95% CI: 19.947–72.557) were all significantly associated with increased malignancy risk (all P < 0.001). Long-axis and short-axis diameters were also significantly larger in the malignant group (both P < 0.001).

Regarding peripheral blood immune-inflammatory indices, NLR, SII, and SIRI were higher in the malignant group than in the benign group (P < 0.001, P = 0.007, and P = 0.041, respectively). PLR, LMR, and PIV did not differ significantly between the two groups (all P > 0.05). These findings indicate that AI-structured DBT imaging features and selected inflammatory indices were associated with the malignancy risk of breast masses (Table 1).

**TABLE 1:** Baseline characteristics and univariable analysis of breast lesions.

Variable	Overall (n = 401)	Malignant (n = 102)	Benign (n = 299)	Z/chi-square	OR	95% CI	P value
Age (years)	44 (38, 52)	54 (46, 62)	41 (36, 48)	8.192	1.093	1.068–1.118	<0.001
**Lesion side**	​	​	​	**1.809**	​	​	**0.179**
Left breast	203 (50.6%)	58 (56.9%)	145 (48.5%)	​	References	​	​
Right breast	198 (49.4%)	44 (43.1%)	154 (51.5%)	​	0.714	0.454–1.123	0.145
**BI-RADS category**	​	​	​	**147.390**	​	​	**<0.001**
3	202 (50.4%)	17 (16.7%)	185 (61.9%)	​	References	​	​
4 A	97 (24.2%)	16 (15.7%)	81 (27.1%)	​	2.150	1.035–4.465	0.040
4B	35 (8.7%)	16 (15.7%)	19 (6.4%)	​	9.164	3.996–21.018	<0.001
4C	43 (10.7%)	31 (30.4%)	12 (4.0%)	​	28.113	12.245–64.545	<0.001
5	24 (6.0%)	22 (21.6%)	2 (0.7%)	​	119.7	25.910–553.1	<0.001
**Lesion depth**	​	​	​	**7.390**	​	​	**0.025**
Anterior third	45 (11.2%)	4 (3.9%)	41 (13.7%)	​	References	​	​
Middle third	225 (56.1%)	63 (61.8%)	162 (54.2%)	​	3.986	1.371–11.588	0.011
Posterior third	131 (32.7%)	35 (34.3%)	96 (32.1%)	​	3.737	1.247–11.195	0.019
**Mass shape**	​	​	​	**140.485**	​	​	**<0.001**
Oval	254 (63.3%)	23 (22.5%)	231 (77.3%)	​	References	​	​
Round	32 (8.0%)	3 (2.9%)	29 (9.7%)	​	1.039	0.294–3.676	0.953
Irregular	115 (28.7%)	76 (74.5%)	39 (13.0%)	​	19.572	10.993–34.845	<0.001
**Mass density**	​	​	​	**125.436**	​	​	**<0.001**
Equal density	304 (75.8%)	35 (34.3%)	269 (90.0%)	​	References	​	​
High density	97 (24.2%)	67 (65.7%)	30 (10.0%)	​	17.165	9.841–29.938	<0.001
Long-axis diameter (mm)	18 (11, 29)	32 (20, 38)	15 (10, 23)	8.313	1.063	1.045–1.082	<0.001
Short-axis diameter (mm)	15 (9, 23)	24 (18, 30)	12 (8, 19)	8.274	1.079	1.055–1.104	<0.001
**Mass margin**	​	​	​	**151.576**	​	​	**<0.001**
Circumscribed	11 (2.7%)	1 (1.0%)	10 (3.3%)	​	References	​	​
Obscured	295 (73.6%)	32 (31.4%)	263 (88.0%)	​	1.217	0.151–9.819	0.854
Indistinct	34 (8.5%)	20 (19.6%)	14 (4.7%)	​	14.286	1.637–124.6	0.016
Microlobulated	6 (1.5%)	5 (4.9%)	1 (0.3%)	​	50.000	2.559–977.0	0.010
Spiculated	55 (13.7%)	44 (43.1%)	11 (3.7%)	​	40.000	4.617–346.6	<0.001
**Calcification**	​	​	​	**179.707**	​	​	**<0.001**
No calcifications	266 (66.3%)	21 (20.6%)	245 (81.9%)	​	References	​	​
Benign calcifications	37 (9.2%)	6 (5.9%)	31 (10.4%)	​	2.258	0.846–6.024	0.104
Suspicious calcifications	98 (24.4%)	75 (73.5%)	23 (7.7%)	​	38.043	19.947–72.557	<0.001
NLR	2.23 (1.72, 2.92)	2.64 (1.98, 3.52)	2.14 (1.68, 2.71)	4.387	1.339	1.131–1.586	<0.001
PLR	154.70 (121.95, 197.71)	161.80 (126.08, 206.69)	152.78 (120.25, 191.64)	1.499	1.002	0.999–1.006	0.134
LMR	5.12 (3.93, 6.81)	4.77 (3.45, 7.03)	5.25 (4.16, 6.77)	−1.265	1.005	0.996–1.013	0.206
SII	502.39 (384.65, 711.76)	569.49 (432.05, 806.50)	485.90 (370.13, 679.34)	2.719	1.001	1.000–1.001	0.007
PIV	149.32 (88.41, 227.85)	161.23 (93.08, 262.10)	146.47 (87.32, 217.59)	1.042	1.001	1.000–1.002	0.298
SIRI	0.64 (0.42, 0.95)	0.73 (0.43, 1.21)	0.62 (0.41, 0.92)	2.042	1.633	1.151–2.316	0.041

Abbreviations: BI-RADS, breast imaging reporting and data system; CI, confidence interval; LMR, lymphocyte-to-monocyte ratio; NLR, neutrophil-to-lymphocyte ratio; OR, odds ratio; PIV, pan-immune-inflammation value; PLR, platelet-to-lymphocyte ratio; SII, systemic immune-inflammation index; SIRI, systemic inflammation response index. Diameter values are reported in millimetres (mm). ORs, for age and lesion diameters correspond to 1-year and 1-mm increases, respectively; future validation studies may report clinically meaningful increments, such as 10 years or 5 mm, to facilitate interpretation. For continuous variables, Z statistics and P values were obtained from Mann-Whitney U tests, whereas ORs, and 95% CIs, were estimated using separate univariable logistic regression models. For categorical variables, overall P values were obtained using the chi-square test or Fisher exact test, as appropriate, and category-specific ORs, and 95% CIs, were estimated using univariable logistic regression with the indicated reference category. Bold values indicate statistical significance (P < 0.05)

### 3.2 Feature selection

**FIGURE 3:**
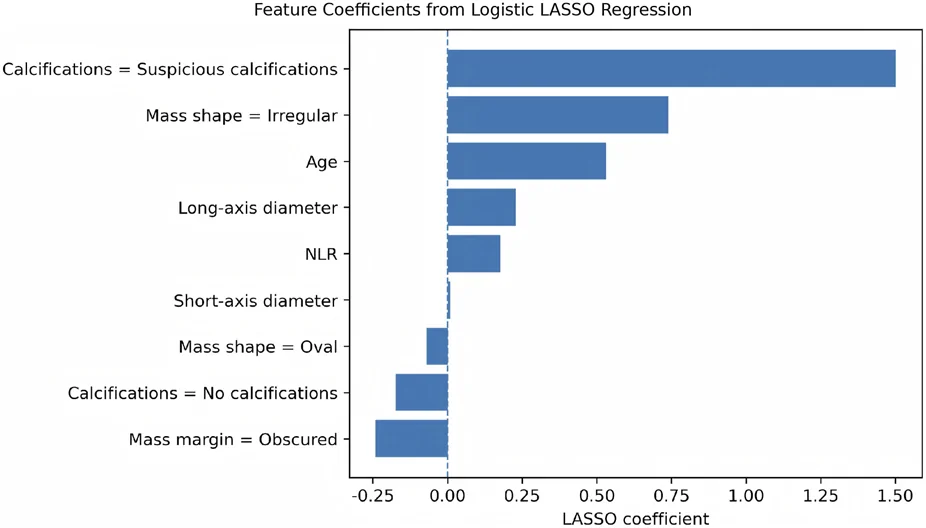
Feature coefficients from logistic LASSO regression. ● Positive coefficients indicate features associated with a higher predicted probability of malignancy, whereas negative coefficients indicate features associated with a lower predicted probability of malignancy.

Logistic LASSO regression with five-fold cross-validation was used for feature selection. The optimal C value was 0.113. Nine nonzero-coefficient features associated with breast cancer prediction were selected: suspicious calcifications, irregular mass shape, age, long-axis diameter, NLR, short-axis diameter, oval mass shape, absence of calcifications, and obscured margin. Suspicious calcifications, irregular mass shape, age, long-axis diameter, NLR, and short-axis diameter had positive coefficients, whereas oval mass shape, absence of calcifications, and obscured margin had negative coefficients. These features were included in subsequent machine-learning model construction (Figure 3).

### 3.3 Model development and validation

Based on the LASSO-selected features, LR, RF, DT, SVM, and XGBoost models were trained and optimized in the training cohort. AUROC was used as the primary performance metric, whereas accuracy, sensitivity, specificity, positive predictive value (PPV), negative predictive value (NPV), F1 score, and Brier score were used as supplementary metrics to evaluate discrimination, classification performance, and probabilistic prediction.

**FIGURE 4:**
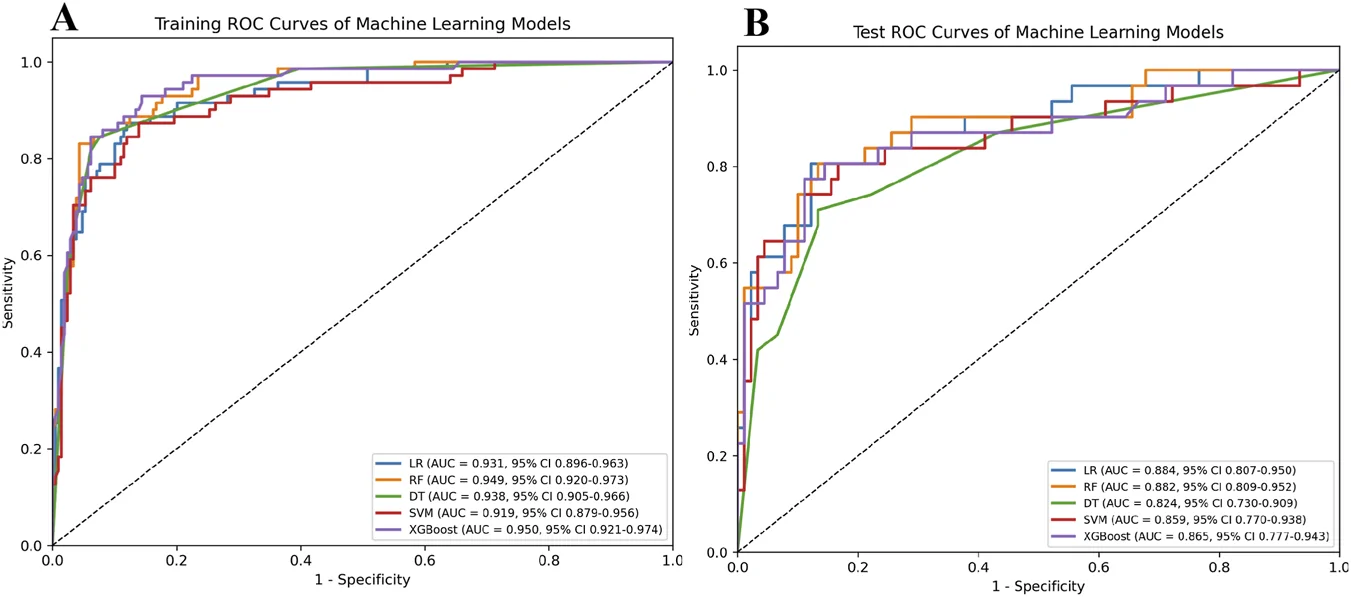
Receiver operating characteristic curves of machine-learning models in the training cohort **(A)** and testing cohort **(B)**. AUROC values and 95% confidence intervals are shown for logistic regression, random forest, decision tree, support vector machine, and XGBoost.

ROC analysis in the training cohort showed that all models had favorable discrimination. XGBoost achieved the highest training AUROC (0.950), followed by RF (0.949), DT (0.938), LR (0.931), and SVM (0.919). In the testing cohort, the LR model achieved the highest AUROC (0.884, 95% CI: 0.807–0.950), followed closely by RF (0.882, 95% CI: 0.809–0.952), XGBoost (0.865, 95% CI: 0.777–0.943), SVM (0.859, 95% CI: 0.770–0.938), and DT (0.824, 95% CI: 0.730–0.909) (Figure 4).

The LR model showed favorable overall classification performance in the testing cohort, with an accuracy of 0.860, sensitivity of 0.806, specificity of 0.878, PPV of 0.694, NPV of 0.929, F1 score of 0.746, and Brier score of 0.118. Although its AUROC was numerically the highest, the difference from RF was minimal (0.884 vs. 0.882), their confidence intervals overlapped, and SVM had a slightly lower Brier score (0.113). Therefore, LR was selected as the final model on the basis of the combined consideration of discrimination, probabilistic performance, clinical applicability, parsimony, and interpretability (Table 2).

**TABLE 2:** Performance of machine-learning models in the primary lesion-level testing cohort and patient-grouped sensitivity analysis.

Model	Testing AUROC (95% CI)	Accuracy	Sensitivity	Specificity	PPV	NPV	F1 score	Brier score	Patient-grouped AUROC (patient-cluster bootstrap 95% CI)	Paired DeLong *P* value vs. LR	Holm-adjusted P
LR	0.884 (0.807–0.950)	0.860	0.806	0.878	0.694	0.929	0.746	0.118	0.884 (0.803–0.955)	References	References
RF	0.882 (0.809–0.952)	0.826	0.581	0.911	0.692	0.863	0.632	0.121	0.871 (0.782–0.945)	0.537	0.652
XGBoost	0.865 (0.777–0.943)	0.818	0.806	0.822	0.610	0.925	0.694	0.134	0.861 (0.766–0.942)	0.326	0.652
SVM	0.859 (0.770–0.938)	0.818	0.742	0.844	0.622	0.905	0.676	0.113	0.859 (0.762–0.942)	0.013	0.052
DT	0.824 (0.730–0.909)	0.826	0.710	0.867	0.647	0.897	0.677	0.154	0.813 (0.719–0.895)	0.02	0.060

Abbreviations: AUROC, area under the receiver operating characteristic curve; CI, confidence interval; DT, decision tree; LR, logistic regression; NPV, negative predictive value; PPV, positive predictive value; RF, random forest; SVM, support vector machine.

In the patient-grouped sensitivity analysis, the development cohort comprised 267 patients with 280 lesions and the testing cohort comprised 115 patients with 121 lesions; no patient appeared in both cohorts. Nineteen patients contributed more than one lesion, including 13 in the development cohort and 6 in the testing cohort. By comparison, a conventional lesion-level random split using the same seed would have placed 8 patients in both cohorts. The LR model retained a testing AUROC of 0.884 (patient-cluster bootstrap 95% CI, 0.803–0.955), with an accuracy of 0.860, sensitivity of 0.806, specificity of 0.878, PPV of 0.694, NPV of 0.929, F1 score of 0.746, and Brier score of 0.118. The comparator AUROCs were 0.871 for RF, 0.861 for XGBoost, 0.859 for SVM, and 0.813 for DT. The unadjusted DeLong P values for LR versus RF, XGBoost, SVM, and DT were 0.537, 0.326, 0.013, and 0.020, respectively; after Holm adjustment, the corresponding P values were 0.652, 0.652, 0.052, and 0.060. Thus, none of the pairwise comparisons remained statistically significant after multiplicity adjustment, and LR was not statistically superior to any comparator (Table 2).

**FIGURE 5:**
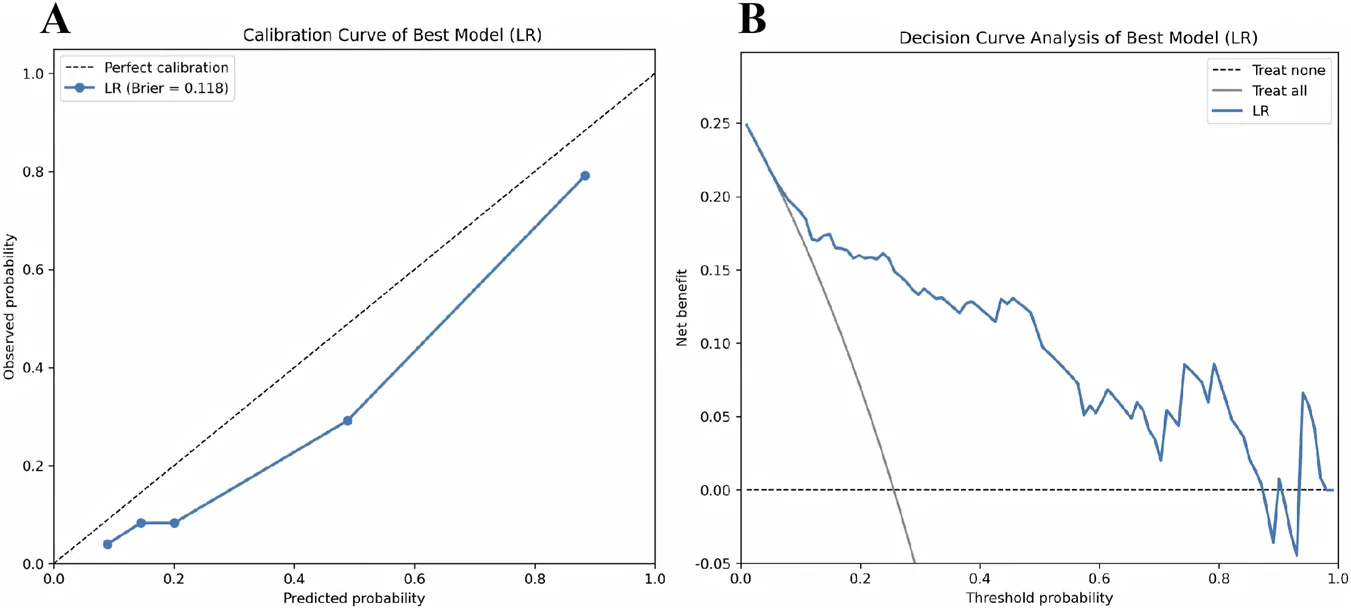
Calibration curve **(A)** and decision curve analysis **(B)** of the final logistic regression model in the testing cohort. The calibration curve shows agreement between predicted probabilities and observed malignancy rates, with the dashed line representing ideal calibration and the Brier score shown in the legend. Calibration intercept and slope were not estimated. Decision curve analysis shows that the model had greater net benefit than the treat-all and treat-none strategies across an approximate threshold-probability range of 0.10–0.85.

**FIGURE 6:**
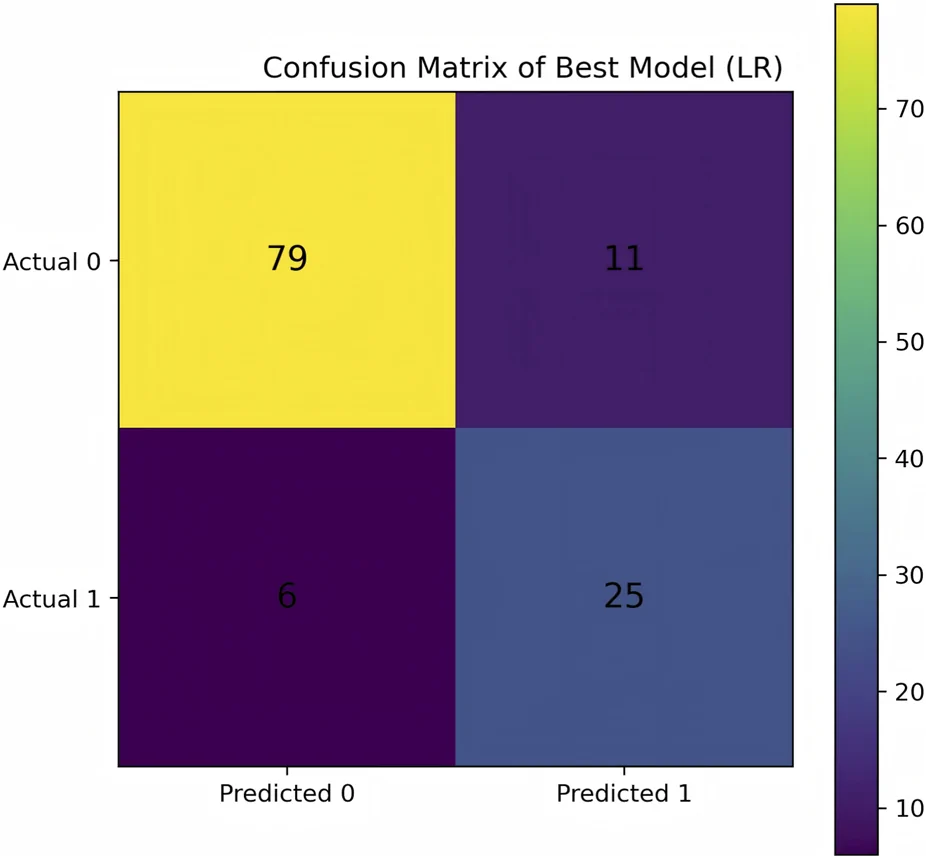
Confusion matrix of the final logistic regression model in the testing cohort. The matrix shows true-negative, false-positive, false-negative, and true-positive classifications at the selected decision threshold. Class 0 indicates benign lesions, and class 1 indicates malignant lesions.

The calibration curve included a dashed ideal-calibration line and yielded a Brier score of 0.118 (Figure 5A). Calibration intercept and slope were not estimated in this exploratory analysis and should be evaluated during future external validation. DCA indicated that the LR model provided greater net benefit than both the treat-all and treat-none strategies across an approximate threshold-probability range of 0.10–0.85 (Figure 5B). In the testing cohort, the confusion matrix of the final LR model showed that 79 benign lesions and 25 malignant lesions were correctly classified, whereas 11 benign lesions were incorrectly classified as malignant and 6 malignant lesions were incorrectly classified as benign. The corresponding accuracy, sensitivity, specificity, PPV, and NPV were 86.0%, 80.6%, 87.8%, 69.4%, and 92.9%, respectively (Figure 6). These findings indicate favorable classification performance, particularly a high rule-out value for malignancy.

### 3.4 Inflammatory-index correlation and stability, model interpretability, and malignancy risk stratification

**FIGURE 7:**
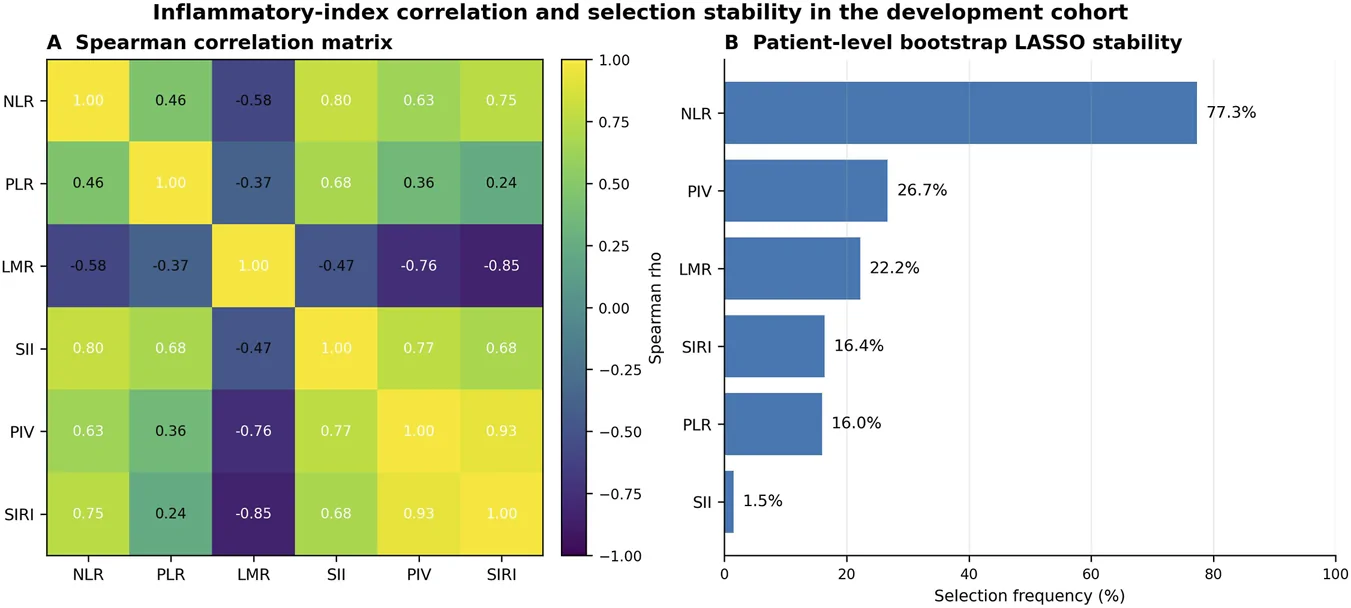
Inflammatory-index correlation and selection stability in the patient-grouped development cohort. **(A)** shows the Spearman correlation matrix of NLR, PLR, LMR, SII, PIV, and SIRI; coefficients displayed in the heatmap are rounded to two decimal places. **(B)** shows the selection frequencies of the six inflammatory indices across 1,000 patient-level bootstrap LASSO resamples; all lesions belonging to a sampled patient were retained together.

In the patient-grouped development cohort, the derived inflammatory indices showed substantial correlation because they share blood-count components. The largest absolute Spearman coefficients were observed for PIV-SIRI (rho = 0.928), LMR-SIRI (rho = −0.854), NLR-SII (rho = 0.800), and SII-PIV (rho = 0.772). In 1,000 patient-level bootstrap samples, NLR was selected in 77.3% of resamples, compared with 26.7% for PIV, 22.2% for LMR, 16.4% for SIRI, 16.0% for PLR, and 1.5% for SII. Thus, NLR had the highest selection frequency in this dataset, although it was not statistically independent of the other derived indices (Figure 7, panels A and B).

**FIGURE 8:**
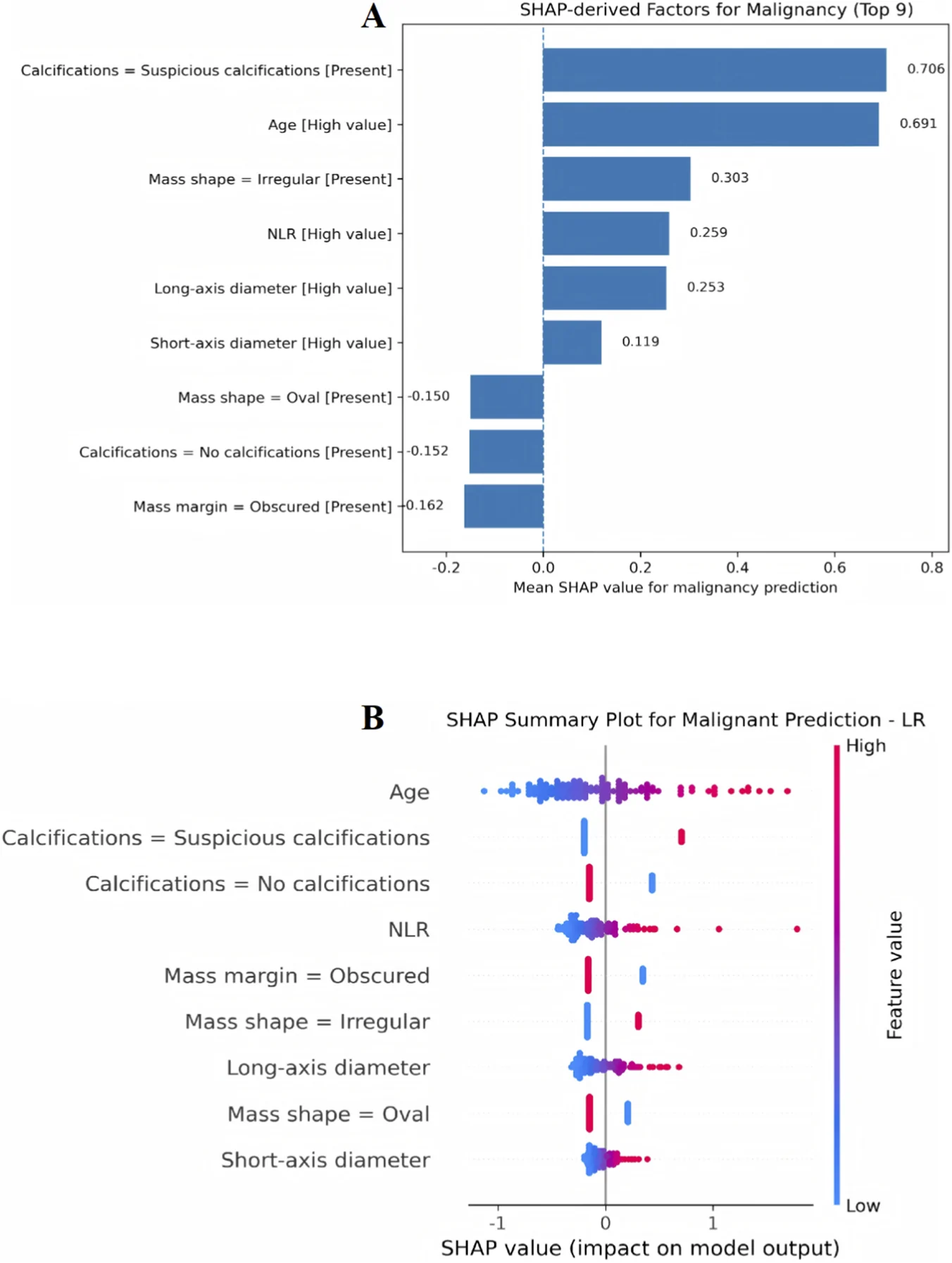
SHAP-derived feature contributions **(A)** and SHAP summary plot **(B)** for malignancy prediction. Figure A shows directional mean SHAP values for selected feature states, and figure B shows the SHAP summary plot for malignancy prediction. Positive SHAP values indicate an increased predicted probability of malignancy, whereas negative SHAP values indicate a decreased predicted probability.

SHAP analysis was used to assess the contribution of each feature to the output of the final LR model. The features with the strongest positive contributions to malignant prediction were suspicious calcifications, older age, irregular mass shape, elevated NLR, larger long-axis diameter, and larger short-axis diameter. Suspicious calcifications and older age had the highest mean SHAP contribution values (0.706 and 0.691, respectively), indicating that they were important drivers of malignant prediction. Irregular mass shape, elevated NLR, and larger long-axis diameter also contributed positively. In contrast, oval mass shape, absence of calcifications, and obscured margin showed negative contributions, suggesting an association with a lower predicted probability of malignancy (Figure 8).

**FIGURE 9:**
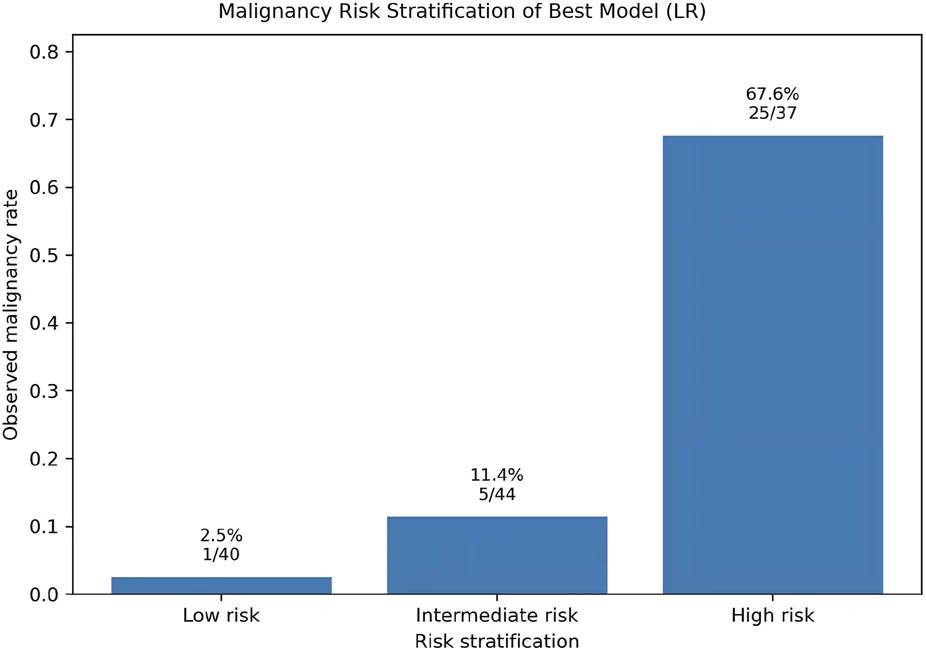
Malignancy risk stratification based on predicted probabilities generated by the final logistic regression model in the testing cohort. Lesions were divided into low-, intermediate-, and high-risk groups according to tertiles of predicted probability. The observed malignancy rate increased progressively across the three groups, from 2.5% (1/40) in the low-risk group to 11.4% (5/44) in the intermediate-risk group and 67.6% (25/37) in the high-risk group.

According to predicted probabilities generated by the LR model in the testing cohort, lesions were categorized into low-, intermediate-, and high-risk groups using cohort-specific tertiles. Among 40 lesions in the low-risk group, 1 was malignant (2.5%). Among 44 lesions in the intermediate-risk group, 5 were malignant (11.4%). Among 37 lesions in the high-risk group, 25 were malignant (67.6%). The observed breast cancer detection rate increased progressively across the predicted risk strata (Figure 9). These tertile cutoffs were used only to demonstrate a gradient of risk and are not intended for direct clinical application.

## 4 Discussion

This study developed an interpretable machine-learning model for benign-malignant differentiation and malignancy risk stratification of breast masses by integrating AI-structured DBT mass features, clinical variables, and peripheral blood immune-inflammatory indices. In the primary testing cohort, all five algorithms demonstrated useful discrimination. The LR model achieved an AUROC of 0.884, which was below 0.90 and only marginally higher than the RF AUROC of 0.882. In the patient-grouped sensitivity analysis, the unadjusted DeLong P values for LR versus RF, XGBoost, SVM, and DT were 0.537, 0.326, 0.013, and 0.020, respectively. After Holm correction, the corresponding P values were 0.652, 0.652, 0.052, and 0.060; therefore, none of the pairwise comparisons remained statistically significant. Conventional DeLong inference is not cluster-robust and was interpreted as supportive. Accordingly, the findings do not establish statistical superiority of LR over any comparator. LR was retained because its discrimination, calibration-related performance, DCA, classification metrics, parsimony, and interpretability were jointly considered. The observed malignancy rates increased from 2.5% to 67.6% across the descriptive risk strata, supporting potential value for individualized risk assessment while also emphasizing the exploratory nature of the present single-center analysis.

SHAP analysis further elucidated the key drivers of malignancy prediction. Suspicious calcifications, advanced age, irregular tumor shape, an elevated neutrophil-to-lymphocyte ratio (NLR), a larger long-axis diameter, and a larger short-axis diameter all contribute positively to the prediction of malignancy. Previous studies have shown that suspicious microcalcifications (especially when accompanied by a mass) have a higher positive predictive value for malignant lesions and are more likely to be associated with aggressive disease (Hegde et al., 2025; Burnside et al., 2007). Hofvind et al. also reported that fine polymorphic calcifications, fine linear calcifications, or fine linear branched calcifications—especially when distributed in clusters or segments—are closely associated with well-differentiated ductal carcinoma *in situ* (Hofvind et al., 2011). This underscores the importance of suspicious calcifications in distinguishing between benign and malignant breast masses and demonstrates that AI-based features derived from structured digital breast tomosynthesis (DBT) can capture clinically relevant radiological information associated with the risk of malignancy.

Age is another important clinical factor in this model. Studies have shown that when patients are older or the lesion exhibits suspicious radiological features (such as calcifications, non-parallel orientation, ill-defined margins, or posterior shadowing), a biopsy is recommended rather than follow-up (Pfob et al., 2022). Even within the same BI-RADS category, the detection rate of breast cancer may increase with age; combining age with BI-RADS assessment can improve diagnostic performance (Deng J. et al., 2024). By integrating clinical and radiological variables into a single framework, this model better reflects the diagnostic reasoning process used in real-world breast imaging practice.

Irregular mass shape was also an important contributor to malignant prediction. This finding is consistent with previous studies showing that irregular shape, nonparallel orientation, angular or spiculated margins, architectural distortion, and vascular signals are associated with higher malignancy risk (Hong et al., 2005; Badu-Peprah et al., 2024). In this study, irregular shape was retained during LASSO feature selection and had a positive contribution in SHAP analysis, indicating that AI-structured DBT-derived morphologic descriptors provide important information for breast mass classification.

NLR showed a positive contribution in both LASSO selection and SHAP analysis, suggesting that systemic immune-inflammatory status may provide complementary information beyond imaging features. NLR is a simple and low-cost biomarker, but the existing breast-cancer literature has focused predominantly on prognostic or treatment-predictive applications rather than benign-malignant differential diagnosis (Yang et al., 2025; Gago-Dominguez et al., 2020). Its diagnostic utility therefore remains emerging, and the present finding should be considered hypothesis-generating. In the patient-grouped development cohort, the supplementary correlation analysis confirmed that NLR, SII, SIRI, PIV, PLR, and LMR were not independent, while patient-level bootstrap analysis showed that NLR had the highest selection frequency among the inflammatory indices (77.3%). This relative stability does not establish a unique biological role, because L1 regularization may retain one representative from a correlated group. Moreover, NLR is not tumor-specific and can be altered by infection, non-neoplastic inflammation, hematologic status, medications, metabolic conditions, and other systemic factors. It should consequently be interpreted only in combination with imaging findings and clinical context, rather than as a standalone diagnostic indicator.

Recent oncology studies further support the value of combining interpretable AI with multimodal biological information. AI-assisted assessment of HER2-positive breast cancer and explainable biomarker modeling illustrate how transparent algorithms may complement conventional clinical interpretation (Deng et al., 2024a; Dogra and Hasija, 2025). Radiogenomic integration that maps gene-expression profiles to image representations also demonstrates the broader potential of linking imaging phenotypes with molecular data (Kawahara et al., 2025). In breast cancer, extracellular-vesicle signaling within the tumor microenvironment highlights biological pathways that may contribute information beyond local morphology (Jahangiri, 2025), while emerging noncoding-RNA mechanisms, including PIWI-interacting RNAs, emphasize the molecular complexity of cancer progression (Deng et al., 2024b). These studies support the rationale for multimodal integration, although the present model remains a diagnostic, hypothesis-generating tool rather than a substitute for radiological or pathological assessment.

From a clinical perspective, one potential value of this model is descriptive risk stratification. The observed malignancy rate was 2.5% in the low-risk group and 67.6% in the high-risk group, and the high NPV suggests possible rule-out value at low predicted probabilities. However, the tertile-based cutoffs are dataset-dependent and should not be used as fixed clinical thresholds. The model was not directly compared with a BI-RADS-only strategy, and net reclassification improvement (NRI) and integrated discrimination improvement (IDI) were not assessed. Therefore, claims regarding biopsy reduction or workflow improvement should be considered provisional. Future studies should establish clinically meaningful thresholds and prospectively compare the integrated model with standard radiologist/BI-RADS assessment.

This study has several limitations. First, it was a single-center retrospective study with a relatively limited sample size, and inclusion required pathologically confirmed lesions. The resulting diagnostically enriched cohort may be affected by selection and spectrum bias and may not represent screening, surveillance, or community populations. Second, lesions rather than patients were used as the unit of analysis in the primary analysis, and the primary split was performed at the lesion level. A supplementary patient-grouped analysis prevented the same patient from entering both cohorts and produced similar LR performance; however, lesions remained the prediction unit and residual within-patient dependence was not modeled using a hierarchical or cluster-robust approach. Third, validation was internal; repeated cross-validation, temporal validation, and external validation were not performed. Pairwise DeLong tests were added only within the supplementary patient-grouped analysis. These conventional lesion-level tests were not cluster-robust, and no comparison remained statistically significant after Holm adjustment; the reported performance should therefore remain exploratory. Fourth, NLR, SII, SIRI, and related indices share blood-count components and were correlated. Although Spearman and bootstrap analyses were restricted to the patient-grouped development cohort, they were *post hoc*, and the stability and biological specificity of NLR require external confirmation. Fifth, calibration intercept and slope were not estimated, and no BI-RADS-only comparator, NRI, or IDI analysis was available. The tertile-based risk groups were also data-dependent and are not clinical cutoffs. Sixth, AI-structured DBT features were derived from a specific imaging device and AI system, and feature extraction may differ across platforms and institutions. Finally, immune-inflammatory markers are influenced by non-neoplastic conditions. Prospective multicentre external validation is required to assess generalizability, calibration, clinical thresholds, and whether the model improves decision-making or reduces unnecessary procedures.

## 5 Conclusion

This study constructed an interpretable LR model integrating AI-structured DBT mass features, clinical variables, and peripheral blood immune-inflammatory indices for benign-malignant differentiation and descriptive malignancy risk stratification. The model demonstrated favorable discrimination and clinically interpretable feature contributions in the internal testing cohort, but it should be regarded as an exploratory decision-support model rather than a replacement for radiologist assessment or pathology. Its stability, calibration, clinical thresholds, and generalizability require prospective multicentre external validation.

## Data Availability

The original contributions presented in the study are included in the article/supplementary material, further inquiries can be directed to the corresponding author.
